# Mechanisms of the Antifungal Action of Marine Metagenome-Derived Peptide, MMGP1, against *Candida albicans*

**DOI:** 10.1371/journal.pone.0069316

**Published:** 2013-07-02

**Authors:** Muthuirulan Pushpanathan, Paramasamy Gunasekaran, Jeyaprakash Rajendhran

**Affiliations:** Department of Genetics, Centre for Excellence in Genomic Sciences, School of Biological Sciences, Madurai Kamaraj University, Madurai, Tamil Nadu, India; University of Wisconsin Medical School, United States of America

## Abstract

**Background:**

Development of resistant variants to existing antifungal drugs continues to be the serious problem in *Candida albicans*-induced fungal pathogenesis, which has a considerable impact on animal and human health. Identification and characterization of newer drugs against *C. albicans* is, therefore, essential. MMGP1 is a direct cell-penetrating peptide recently identified from marine metagenome, which was found to possess potent antifungal activity against *C. albicans*.

**Methodology/Principal Findings:**

In this study, we investigated the mechanism of antifungal action of MMGP1 against *C. albicans*. Agarose gel shift assay found the peptide to be having a remarkable DNA-binding ability. The modification of the absorption spectra and fluorescence quenching of the tryptophyl residue correspond to the stacking between indole ring and nucleotide bases. The formation of peptide–DNA complexes was confirmed by fluorescence quenching of SYTO 9 probe. The interaction of peptide with plasmid DNA afforded protection of DNA from enzymatic degradation by DNase I. *In vitro* transcription of mouse β-actin gene in the presence of peptide led to a decrease in the level of mRNA synthesis. The *C. albicans* treated with MMGP1 showed strong inhibition of biosynthetic incorporation of uridine analog 5-ethynyluridine (EU) into nascent RNA, suggesting the peptide’s role in the inhibition of macromolecular synthesis. Furthermore, the peptide also induces endogenous accumulation of reactive oxygen species (ROS) in *C. albicans*. MMGP1 supplemented with glutathione showed an increased viability of *C. albicans* cells. The hyper-produced ROS by MMGP1 leads to increased levels of protein carbonyls and thiobarbituric acid reactive substances and it also causes dissipation of mitochondrial membrane potential and DNA fragmentation in *C. albicans* cells.

**Conclusion:**

**And Significance**: Therefore, the antifungal activity of MMGP1 could be attributed to its binding with DNA, causing inhibition of transcription followed by endogenous production of ROS, which triggers cascade of events that leads to cell death.

## Introduction

Fungal disease, in a majority of the cases, in immunocompromised individuals is caused by the opportunistic fungal pathogen, *Candida albicans*. The increasing incidence of candidiasis and a rapid acquisition of drug resistance by *C. albicans* to the conventional antifungal drugs necessitate the development of novel anticandidal drug [1]. The therapy involving antimicrobial peptides (AMPs) has received greater attention in recent years as pathogenic microorganisms are less likely to develop resistance to these peptides [2].

The mechanism of action of antifungal peptides involves membrane permeabilization/translocation, inhibition of cellular processes and formation of reactive oxygen species (ROS) in the fungal species [3]. AMPs have been known to translocate across the cell membrane and exert antimicrobial action by affecting macromolecular processes such as protein, nucleic acid and cell wall synthesis and enzyme activities [4]. The formation of ROS has been suggested to play a pivotal role in the fungicidal activity of most of the antifungal peptides. Certain antifungal peptides such as papillocin [5], mellitin [6], histatin 5 [7], lactoferrin [8], pleurocidin [9] exhibit anticandidal activity by inducing apoptosis. Fungicidal activity of other antifungal peptides such as LL-37 [10], PAF26 [11], lactoferrin and histatin5 [12] is caused by their binding with nucleic acids. Recently, in our laboratory, we have identified a novel peptide [MLWSASMRIFASAFSTRGLGTRMLMYCSLPSRCWRK (MMGP1)] from marine metagenome, which exhibited chitin binding and direct cell-penetrating property and was found to possess potent antifungal activity against *C. albicans* and *A. niger*. The minimum inhibitory concentration of the peptide was reported to be 0.57 µM for *C. albicans* and 4.29 µM for *A. niger* [13,14]. The mechanism of action this peptide in exhibiting antifungal activity remains unexplored. Hence, we investigated the mechanism of antifungal action of MMGP1 in *C. albicans* in this study.

## Materials and Methods

### Ethics Statement

The blood sample for hemolytic activity assay was collected from a healthy individual with written consent after the approval of Internal Research Review Board and Ethical Clearance Committee of Madurai Kamaraj University.

### Peptide synthesis

MMGP1 peptide was synthesized with >98% purity employing solid phase methods using *N*-(9-fluorenyl) methoxycarbonyl (Fmoc) chemistry (Genscript Corporation, Piscataway, NJ, USA). The peptide (5 mg) was dissolved in 1 ml of 50 mM Tris buffer (pH 7.4) and appropriately diluted sample was used for subsequent analysis.

### Cell treatment and microscopic analysis

In all experiments, the *C. albicans* cells were treated with a determined minimum inhibitory concentration (0.57 µM) of MMGP1 for different time intervals at 30°C. The *C. albicans* cells treated with 1 mM of H_2_O_2_ were used as a positive control. For fluorescence microscopic analysis, the treated cells stained with fluorescent probe at respective time intervals were collected by centrifugation at 10,000 × *g* for 10 min; subsequently the cells were washed with phosphate-buffered saline (PBS) and examined under Nikon Eclipse *Ti* fluorescence microscope (Nikon, Tokyo, Japan).

### DNA binding assay

The plasmid DNA Bluescript II SK (+) was purified using a QIAprep Spin Minprep kit (Qiagen, Germay) and used for subsequent analysis. The plasmid DNA (100 ng) was mixed with varying concentrations of peptide such as 0.036, 0.072, 0.144, 0.288 and 0.576 µM, respectively, in 20 µl of HBS (21 mM Hepes-NaOH buffer containing 135 mM NaCl, 5.0 mM KCl and 0.76 mM Na_2_HPO_4,_ pH 7.4) buffer and the mixture was incubated at room temperature for 30 min and then subjected to electrophoresis on 1% agarose gel.

### Fluorescence quenching assays

All fluorescence measurements were carried out in 96-well black bottom microtitre plates using SpectraMax ^®^e microplate reader (Molecular Devices, CA, USA). The changes in the tryptophan fluorescence of MMGP1 on addition of plasmid DNA were recorded using the method described by Hsu et al., (2005) [15]. The plasmid DNA (100 ng) was incubated with varying concentrations of peptide as described previously for 30 min at room temperature. The emission spectra were recorded from 300 to 400 nm with an excitation wavelength of 295 nm. The fluorescence spectra were determined for peptide alone or in the presence of different concentrations of plasmid DNA.

Further, the fluorescence quenching of SYTO 9 was measured at varying concentrations of the peptide as described previously. The plasmid DNA was incubated with varying concentrations of peptide for 30 min at room temperature to allow for the formation of the DNA–peptide complex. SYTO 9 dye was added to the formed complexes at a final concentration of 5 µM and was incubated for 30 min at room temperature under total darkness. After incubation, the fluorescence quenching of SYTO 9 dye was measured at an excitation wavelength of 485 nm and emission wavelength of 498 nm.

### DNase I protection assay

The DNase I protection assays were performed as per the protocol described by Niiodme et al., (1997) [16]. The assay were performed by mixing 100 ng of the plasmid DNA (blue script SK+) with varying concentrations of peptides as described previously, in 45 µl of HBS and incubated for 30 min at room temperature. After incubation, 5 µl solution of 10 mM MgCl_2_ and 10 mM CaCl_2_ were added to the mixture, followed by addition of 5 µl of 0.5 mg/ml DNase I (Sigma Aldrich, CA, USA) in water and the solution was incubated at 42^°^C for 30 min. To dissociate the plasmid DNA bound to the peptide, 2 µl of proteinase K (20 mg/ml) was added and the solution incubated at 55^°^C for 30 min. After incubation, the mixture was electrophoresed on 1% agarose gel.

### *In vitro* transcription experiment

The inhibition of transcription by MMGP1 was studied *in vitro* using MAXIscript T7 *in vitro* transcription kit (Invitrogen, USA). The pTRI-actin control template harbouring mouse β-actin gene with T7 promoter was mixed with varying concentrations of peptide (0.036, 0.072, 0.144, 0.288 and 0.576 µM) for 30 min at room temperature. *In vitro* transcription reactions were performed individually for the control templates treated with different concentrations of peptide for 1 h at 37^°^C. After transcription reaction, 1 µl of TURBO DNase was added to the mixture and it was incubated at 37^°^C for 10 min to remove the control template DNA. The transcribed product of 304 bases were analysed on 5% denaturing polyacrylamide gel. The transcripts level was quantified by gel densitometry analysis using gel imaging system (Bio-Rad, USA).

### *In vivo* transcription assay

The inhibition of transcription by MMGP1 in *C. albicans* was studied based on the biosynthetic incorporation of the uridine analog 5-ethynyluridine (EU) into the newly transcribed RNA as described by Jao and Salic, (2008) [17]. The *C. albicans* cells were grown in potato dextrose broth (PDB) in the presence of EU (1 mM) for 6 h at 30^°^C and subsequently, treated with MMGP1 (0.57 µM) for 0, 2, 6, 12 and 24 h and collected by centrifugation at 10, 000 × *g* for 10 min. The collected cells were rinsed and fixed in PBS with 0.5% formaldehyde and 0.5% Triton-X-100 for 30 min at room temperature. For EU detection, the cells were rinsed with Tris-buffered saline (TBS) and stained with 100 mM Tris (pH 5.8)/1 mM CuSO_4_/25 µM tetramethyl rhodamine-azide (TMR-A)/100 mM ascorbic acid for 30 min at room temperature. After staining, the cells were collected and washed thrice with TBS containing 0.5% Triton X-100 and counter-stained with Hoechst 33342 (5µM) for 30 min at room temperature under darkness. The cells were examined under Operatta High content imaging system (PerkinElmer, Massachusetts, USA).

### ROS imaging and quantification

The endogenous production of ROS in *C. albicans* cells was analyzed after its 6 h treatment with MMGP1 or H_2_O_2_ by 2', 7'-dichlorodihydrofluorescein diacetate (H_2_DCF-DA) staining followed by fluorescence microscopy [18]. For quantitative assessment of ROS production, the population of cells exhibiting dichlorofluorescein (DCF) fluorescence were measured at 1, 3 and 6 h of treatment with the peptide using flow cytometry.

### Viability assay

Time-kill experiment for antifungal activity was performed turbidimetrically with supplementation of glutathione, an antioxidant. The exponentially growing cultures of *C. albicans* were treated with peptide in the presence of different concentrations of glutathione (1, 10 and 50 mM) for 24 h. The absorbance at 600 nm was measured at 6, 12, 18, and 24 h using SpectraMax ^®^e microplate reader. The culture without peptide was used as a control.

### Measurement of protein oxidation

Intracellular oxidation of proteins in *C. albicans* could be determined by measuring the carbonyl groups generated in some amino acid side chains using dinitophenylhydrazine (DNPH) derivatization method [19]. The level of protein carbonyls in MMGP1- or H_2_O_2_-treated cells were measured for 24 h. At every 6 h of treatment, 500 µl of cells were collected and the cell lysates were prepared by ultrasonication. The protein present in the cell lysates were quantified using Lowry’s method. Protein samples (250 µl; 12.4 mg/ml) were incubated with or without DNPH solution (1 ml) for 45 min at room temperature in dark with intermittent gentle mixing. The reaction mixture was added to 1.25 ml of 10% trichloroacetic acid solution and incubated on ice for 10 min. After incubation, the suspension was centrifuged at 10,000 × *g* for 10 min at 4^°^C and the supernatant fraction was discarded. The pellet was washed five times with 1 ml of ethanol/ethyl acetate (1: 1 v/v) and resuspended in 250 µl of 50 mM Tris-HCl (pH 7.4) buffer and incubated for 10 min at 37^°^C. The solubilized protein in the buffer was quantified prior to assay. The supernatant fraction was transferred to a minicuvette and the absorbance was measured at 375 nm using spectrophotometer (Hitachi U-2900, Tokyo, Japan). Protein carbonylation was determined as follows: protein carbonyls (nmol/ml) = A_375nm_ × 45.45 (nmol/ml); protein carbonyl (nmol/mg) = protein carbonyl (nmol/ml) / protein concentration (mg/ml).

### Measurement of lipid peroxidation

The MMGP1-induced oxidation of lipids in *C. albicans* was determined by quantitative measurement of thiobarbituric acid (TBA) – reactive substances (TBARS) [20]. The production of TBARS in MMGP1- or H_2_O_2_-treated cells was measured for 24 h. At every 6 h of treatment, 500 µl of cell suspension was removed and the cells were collected by centrifugation at 10,000 × *g* for 10 min. The cell pellet was washed twice with 500 µl of sterile distilled water and resuspended in same volume of sterile distilled water. To the cell suspension, 1 ml of TBA reagent (0.25 M HCl, 15% [w/vol] trichloroacetic acid, 0.375% [w/vol] TBA) was added and the reaction was terminated. The mixture was boiled at 100°C for 15 min in a water bath and allowed to cool at room temperature. Cell debris was removed by centrifugation at 3000 × *g* for 5 min and the TBARS was assayed in the supernatant at 535 nm. TBA reagent mixed with 0.5 ml of distilled water was used as the blank. The concentration of TBARS in the samples was determined against the reference tetraethoxypropane.

### Determination of mitochondrial membrane potential

The mitochondrial membrane potential in MMGP1-treated *C. albicans* cells were analyzed by rhodamine 123 staining followed by flow cytometry analysis. Rhodamine 123 is a cell-permeant cationic fluorescent dye sequestered by active mitochondria. The fluorescence quenching of rhodamine 123 directly depends upon the electrochemical gradient across the mitochondrial membrane [21]. Mitochondrial membrane potential in MMGP1-treated *C. albicans* cells was measured for 24 h. At every 6 h of treatment, the cells were collected by centrifugation at 10,000 × *g* for 10 min and subsequently stained with 100 nM of Rhodamine 123. The population of cells exhibiting green fluorescence was quantified using flow cytometry.

Further, the depolarization of inner mitochondrial membrane in MMGP1-treated *C. albicans* cells was assessed by cardiolipin-specific nonyl acridine orange (NAO) staining [22]. The *C. albicans* cells were grown in 500 ml of potato dextrose broth in the presence or absence of peptide for 24 h at 30°C. The treated and untreated cells were collected by centrifugation at 500 × *g* for 10 min; subsequently the cells were washed twice with distilled water and once with 1 M sorbitol. The cells were resuspended in 5 ml of spheroplasting buffer (1 M sorbitol, 25 mM EDTA, 100 mM Na-citrate [pH 5.8]) along with lyticase (2.5 mg per g [wet weight] of yeast cells) and incubated for 2 h at 30°C with gentle shaking. The osmotically sensitive cells were lysed using 10% sodium dodecyl sulphate (SDS). The spheroplast obtained was centrifuged at 500 × *g* for 5 min and washed twice with 1 M sorbitol. The cells were then resuspended in 5 ml of ice cold breaking buffer (0.6 M sorbitol, 20 mM HEPES, 1 mM EDTA, 1 mM phenylmethylsulfonyl fluoride [pH 7.4]) and homogenized in a glass homogenizer on ice. The homogenized mixture was centrifuged at 500 x *g* for 5 min at 4°C and the pellet fraction was discarded. The supernatant fractions containing mitochondria were collected and centrifuged at 12,000 × *g* for 15 min at 4°C, and the pellet fraction was resuspended in osmotic buffer (10 mM Tris-HCl, 0.6 M mannitol, 2 mM EGTA [pH 6.8]). The mitochondria in osmotic buffer were stained with NAO for 20 min in total darkness at room temperature. The fluorescence quenching of NAO by mitochondria was visualized under fluorescence microscope at an excitation wavelength of 485 nm and emission wavelength of 530 nm.

### DNA fragmentation assay

The MMGP1-induced DNA damage in *C. albicans* was assessed by terminal deoxynucleotidyl transferase (TdT)-mediated dUTP nick-end labelling (TUNEL) method. The *C. albicans* cells were treated with MMGP1 (0.57 µM) for 0, 6, 12 and 24 h. The cells treated with H_2_O_2_ were used as positive control. The MMGP1- or H_2_O_2_-treated cells were fixed in 4% paraformaldehyde for 10 min at room temperature and permeabilized with 200 µl of 70% ice-cold ethanol. The permeabilized cells were washed twice with 200 µl of PBS for 5 min at room temperature. The washed cells were resuspended in 50 µl of TdT equilibration buffer and incubated at 37^°^C for 10 min with occasional gentle mixing. After incubation, the cells were collected by centrifugation at 10,000 × *g* for 10 min and washed with 200 µl of PBS. The cells were resuspended in 100 µl TdT staining buffer containing fluorescein-12-dUTP (40 pmol/µl) and TdT enzyme (0.5 U/µl) and incubated at room temperature for 30 min in dark. The stained cells were collected by centrifugation and washed twice with 200 µl of PBS. The washed cells were resuspended in 50 µl of PBS and counter-stained with Hoechst stain 33342 (5µM) for 30 min at room temperature under darkness. After staining, the cells were washed with 50 µl of PBS for 5 min at room temperature and mounted in glycerol/PBS (9:1, v/v) solution containing 0.1% *p*-phenylendiamine dihydrochloride. The nuclei of the treated cells were then examined under Operetta High Content Imaging System. The population of cells positive for TUNEL were quantified by flow cytometry.

### Hemolytic activity assay

The hemolytic activity of the MMGP1 was determined on 1% human red blood cells. Freshly collected blood cells were washed in PBS (pH 7.4) and finally resuspended in four times their original volume of PBS. A 50 µl of 1% HRBC suspension was aliquoted into wells of 96-well microtiter plates, and the peptide was added to the suspension at a final concentration of 0.37, 0.74, 1.48, 2.96, 5.92, 11.84, 23.68, 47.36 µM to a final volume of 100 µl. Samples were incubated at 37°C for 1 h and centrifuged at 1400 × *g* for 10 min, and the supernatant was transferred to fresh 96-well microtiter plates. The hemoglobin release was monitored by measurement of absorbance at 540 nm with a SpectraMax ^®^e microplate reader. The hemolytic activity of MMGP1 was calculated as the percentage of total hemoglobin released compared with that released by incubation with 0.1% Triton X-100 by using the following formula: % hemolysis = (A540nm in th**e** peptide solution – A540nm in PBS) / (A540nm in 0.1% Triton X-100 – A540nm in PBS) × 100**.**

### Flow cytometry analysis

MMGP1-induced ROS production, mitochondrial membrane damage, DNA damage and transcription inhibition in *C. albicans* was measured by flow cytometry [10,000 cells were gated for ROS induction, mitochondrial and DNA damage experiments, whereas, 50,000 cells were gated for transcription inhibition assay on live cells by Forward scatter (FSC)/Side scatter (SSC)] using fluorescence activated cell sorter (FACSAria III, Beckton Dickinson, San Jose, CA, USA). For measurement of MMGP1-induced ROS production, mitochondrial membrane damage and DNA damage in *C. albicans* cells, the treated cells were stained with H_2_DCF-DA, rhodamine123 and fluorescein-12-dUTP, respectively, at different intervals, for 30 min at room temperature. The stained cells were collected by centrifugation at 10,000 × *g* for 10 min; subsequently the cells were washed with 500 µl of PBS and resuspended in the same volume of PBS. The population of cells exhibiting green fluorescence was quantified using FACS at 488 nm employing a blue laser with emission in the 530⁄30 band pass filter. For *in vivo* transcription inhibition assay, the EU labelled cells after treatment with MMGP1 was stained with TMR - azide as described previously. The population of cells with red fluorescence was quantified using FACS at 630 nm employing a green laser with emission in the 610/20 nm band pass filter. The analysis of the results was carried out using FACSDiva version 6.1.3 software (Beckton Dickinson).

## Results

### DNA-binding ability of MMGP1

**Figure 1:**
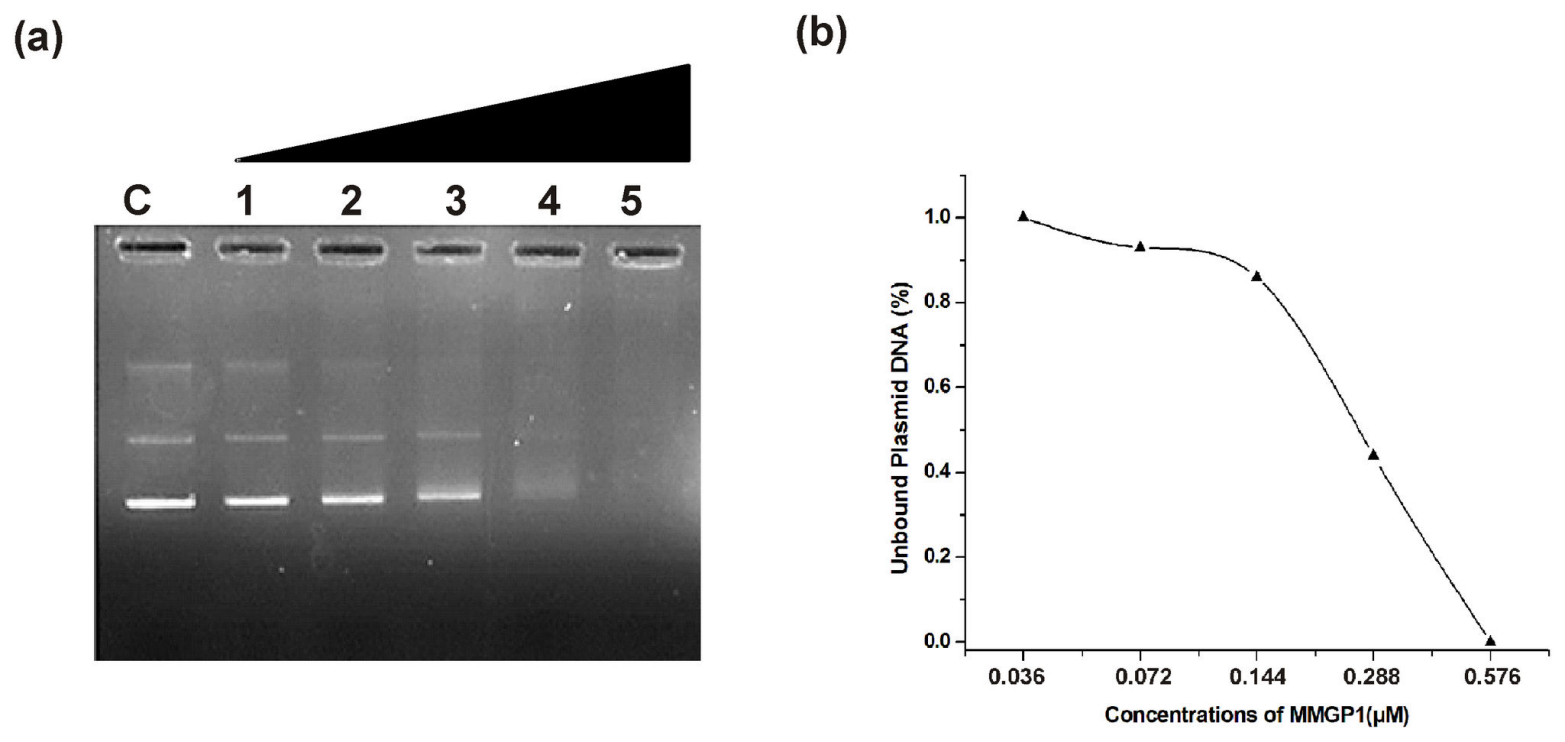
DNA-binding ability of MMGP1. (a) The panel shows the different mixtures of DNA (100 ng) and MMGP1 (1- 0.036; 2- 0.072; 3- 0.144; 4- 0.288; 5-0.576 µM; C-without peptide) run on a 1% agarose gel. (b) Quantitative assessment of unbound plasmid DNA to the peptide by gel densitometry analysis.

The DNA-binding ability of the peptide was evaluated by agarose gel electrophoresis (Figure 1a & 1b). At peptide concentrations of 0.036 and 0.072 µM, the plasmid DNA migrates and stains normally in gel as non-complexed DNA. More than 40% of the plasmid DNA remains unbound at a concentration of 0.288 µM, whereas, at a higher peptide concentration (0. 576 µM), no DNA bands were detected on the gel, showing the intrinsic DNA-binding ability of MMGP1.

### Fluorescence Quenching

**Figure 2:**
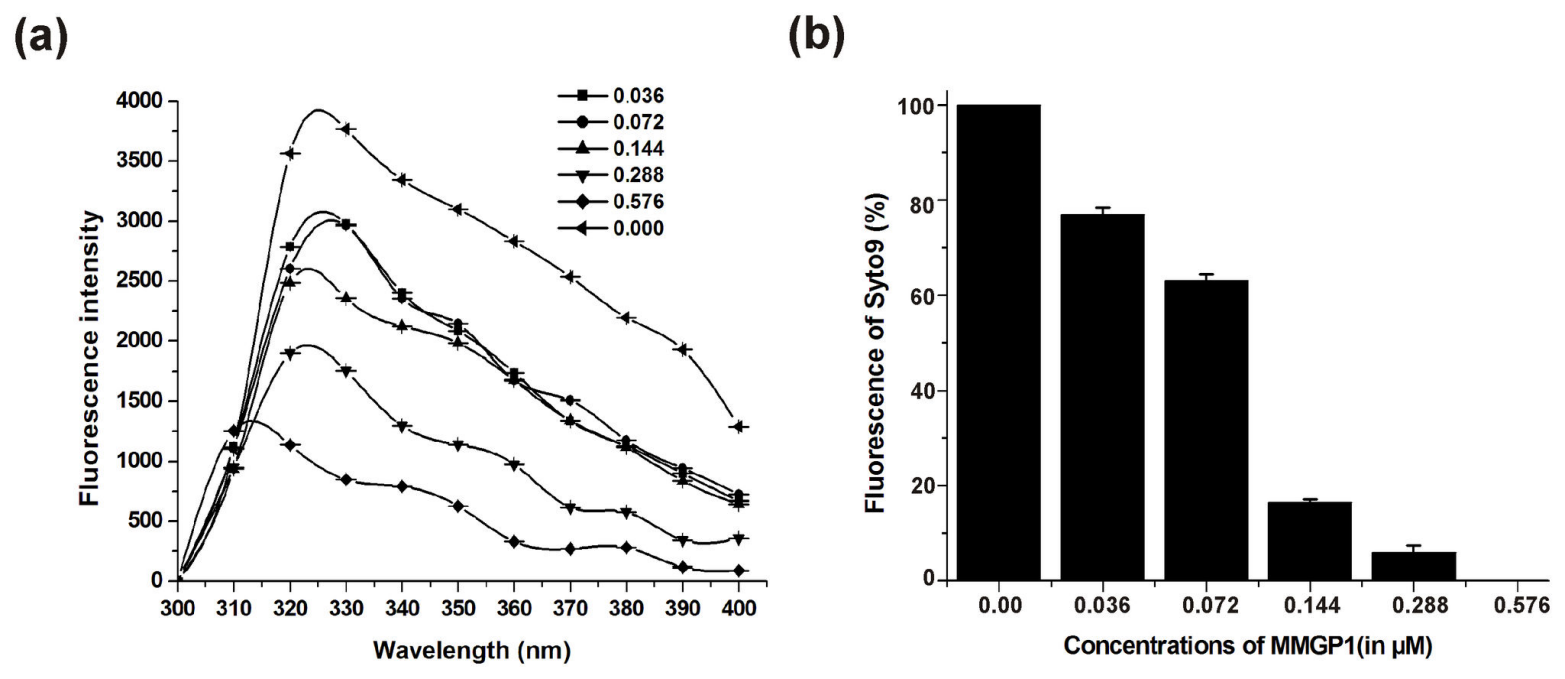
Fluorescence quenching assays for MMGP1 binding to DNA. (a) Measurement of intrinsic tryptophan fluorescence of MMGP1 on addition of plasmid DNA (b) Quenching of SYTO9 fluorescence in the presence of varying concentrations of MMGP1.

The intrinsic tryptophan ﬂuorescence spectra were used to ascertain the conformational changes and the interactions between DNA and MMGP1, since the intensity and the emission maximum depend on the surrounding environment of the indole ring of the tryptophan residue. The MMGP1 peptide contains two tryptophan residues and the DNA binding is expected to change the polarity of the environment around these residues. The modification of fluorescence spectra of tryptophan on the addition of different concentrations of plasmid DNA is shown in Figure 2a. The results revealed that in the absence of DNA, MMGP1 exhibited a fluorescence emission maximum at 330 nm, and addition of DNA caused the fluorescence intensity to decrease and the emission maximum to shift from 330 nm to 310 nm, indicating that the conformational changes had occurred in the environment of tryptophan residues on DNA binding. Furthermore, we also examined the fluorescence quenching of SYTO 9 at varying concentrations of peptide as shown in Figure 2b. The results revealed that in the absence of the peptide, an enhanced fluorescence of the SYTO 9 probe was observed. Fluorescence intensity of SYTO 9 decreased with a further increase in the peptide concentration and at a higher concentration of peptide (0.576 µM), no fluorescence of the probe was observed, which clearly indicates that the binding of peptide with DNA prevents the intercalation of the probe into nucleotide bases.

### DNase I protection assay

**Figure 3:**
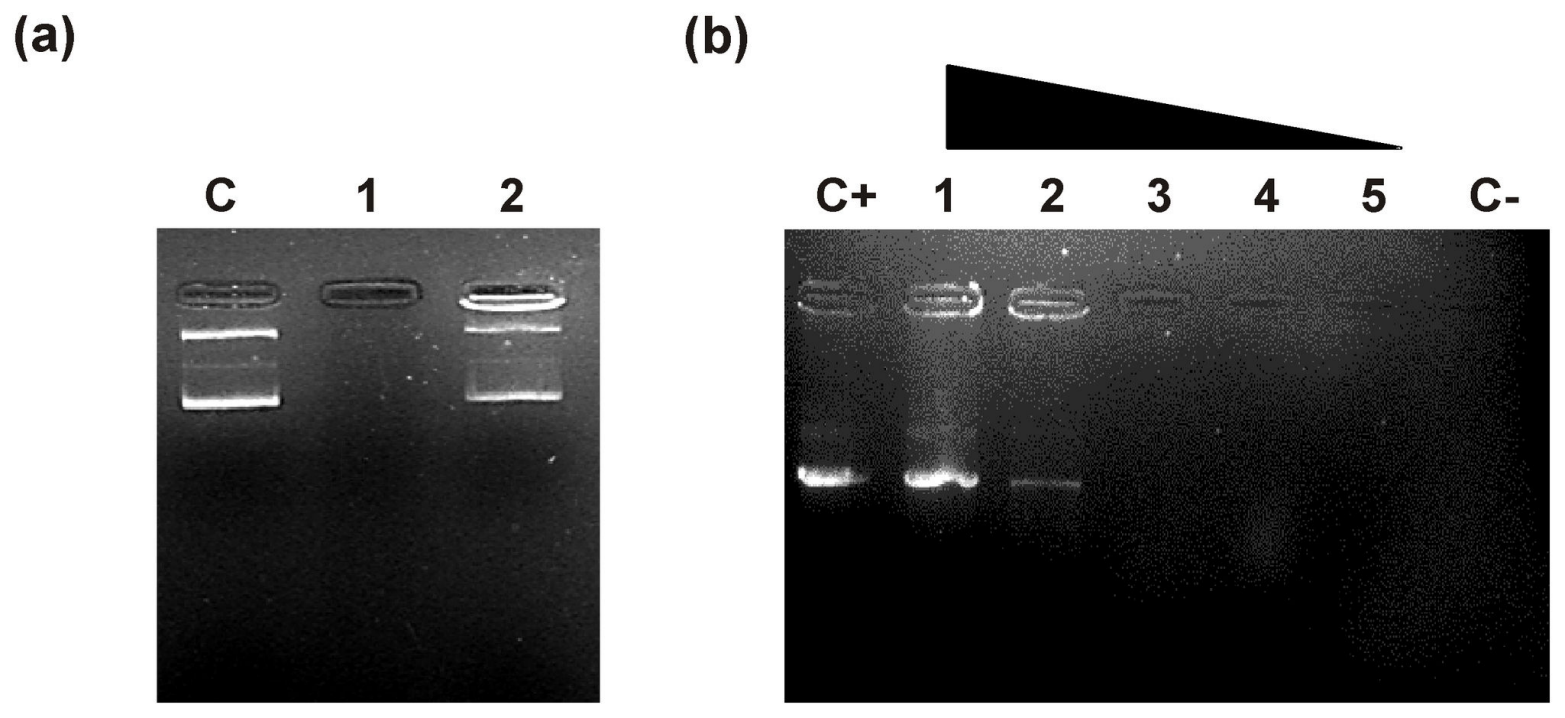
Effect of proteinase K and DNase I on DNA–MMGP1 complexes. (a) Treatment of DNA–MMGP1 complexes with proteinase K resulted in detection of plasmid DNA band on the agarose gel. C-Control SK+ plasmid DNA; 1-DNA–MMGP1 complex formed at 0.576 µM concentration of peptide; 2-DNA–MMGP1 complex treated with proteinase K (b) DNase I protection assay. The plasmid DNA (100 ng) was preincubated with the varying concentrations peptides such as, C+-no peptide; 1-0.576 µM; 2-0.288 µM; 3- 0.144 μM; 4-0.072 µM; 5-0.036 µM, respectively followed by treatment with DNase I. The DNase treated plasmid DNA was used as negative control (C-).

The formation of DNA–peptide complexes was confirmed by treating the formed complexes with proteinase K, which showed detection of bound plasmid DNA in agarose gel (Figure 3a). The DNA-binding ability of the peptide was evaluated by studying the inhibitory activities of the nuclease. The binding of peptide with plasmid DNA affords protection of DNA from nuclease activity as shown in Figure 3b. The DNA bands were prominent on the gel at a peptide concentration of 0.576 µM, whereas a small fraction of plasmid DNA was subjected to nuclease digestion at a peptide concentration of 0.288 µM. However, digestion of DNA by the DNase1 took place without resistance at the lesser peptide concentration, indicating that MMGP1 had the strongest DNA-binding ability to plasmid DNA. These findings were consistent with the results from the DNA-binding assay as described above.

### Transcription inhibition by MMGP1

**Figure 4:**
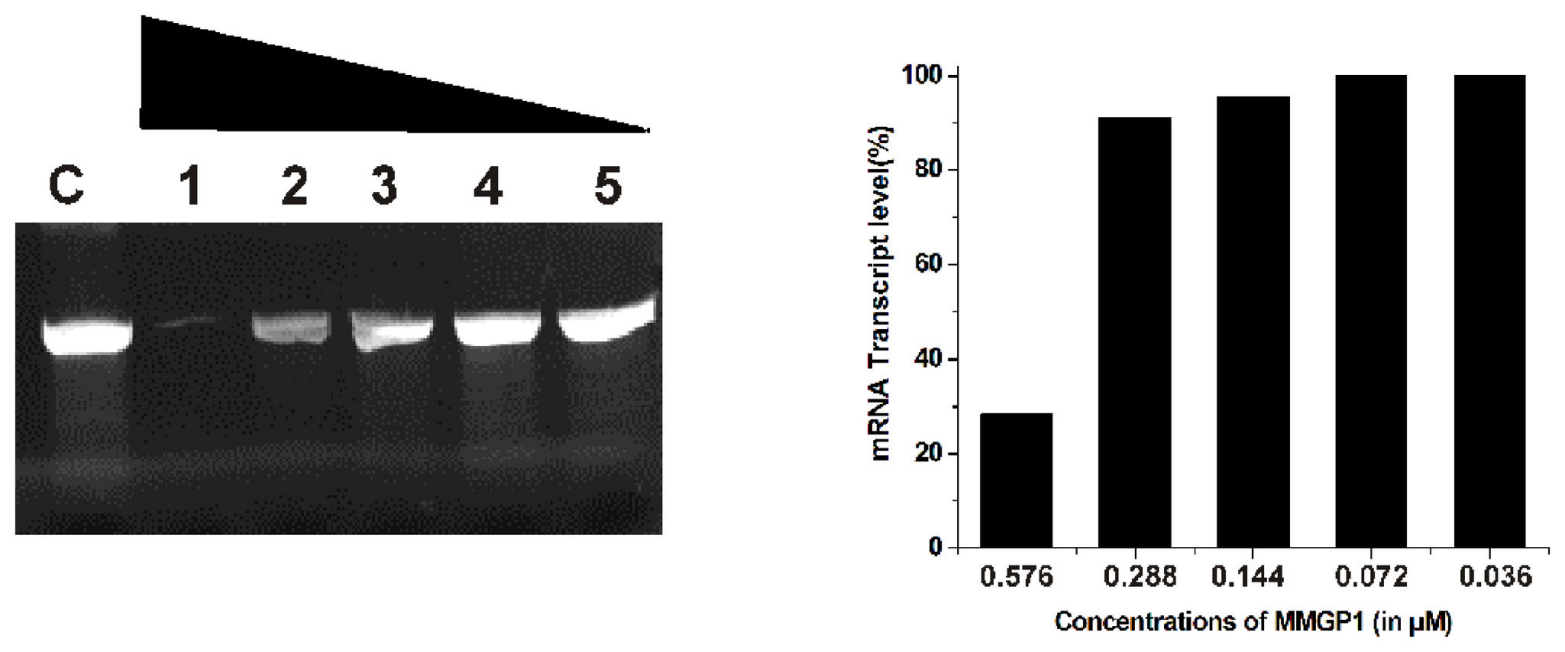
*In vitro* transcription inhibition by MMGP1. (a) Expression of mouse β-actin gene in the presence of different concentrations of MMGP1 C-no peptide, 1-0.576 µM, 2-0.288 µM, 3- 0.144 μM, 4- 0.072; 5- 0.036 at 37 ^°^C for 1 h under *in vitro* condition. The transcribed product of 304 bases were analysed on 5% denaturing polyacrylamide gel. (b) Quantitative measurement of mouse β-actin gene expression in the presence of varying concentrations of MMGP1 by gel densitometry analysis.

**Figure 5:**
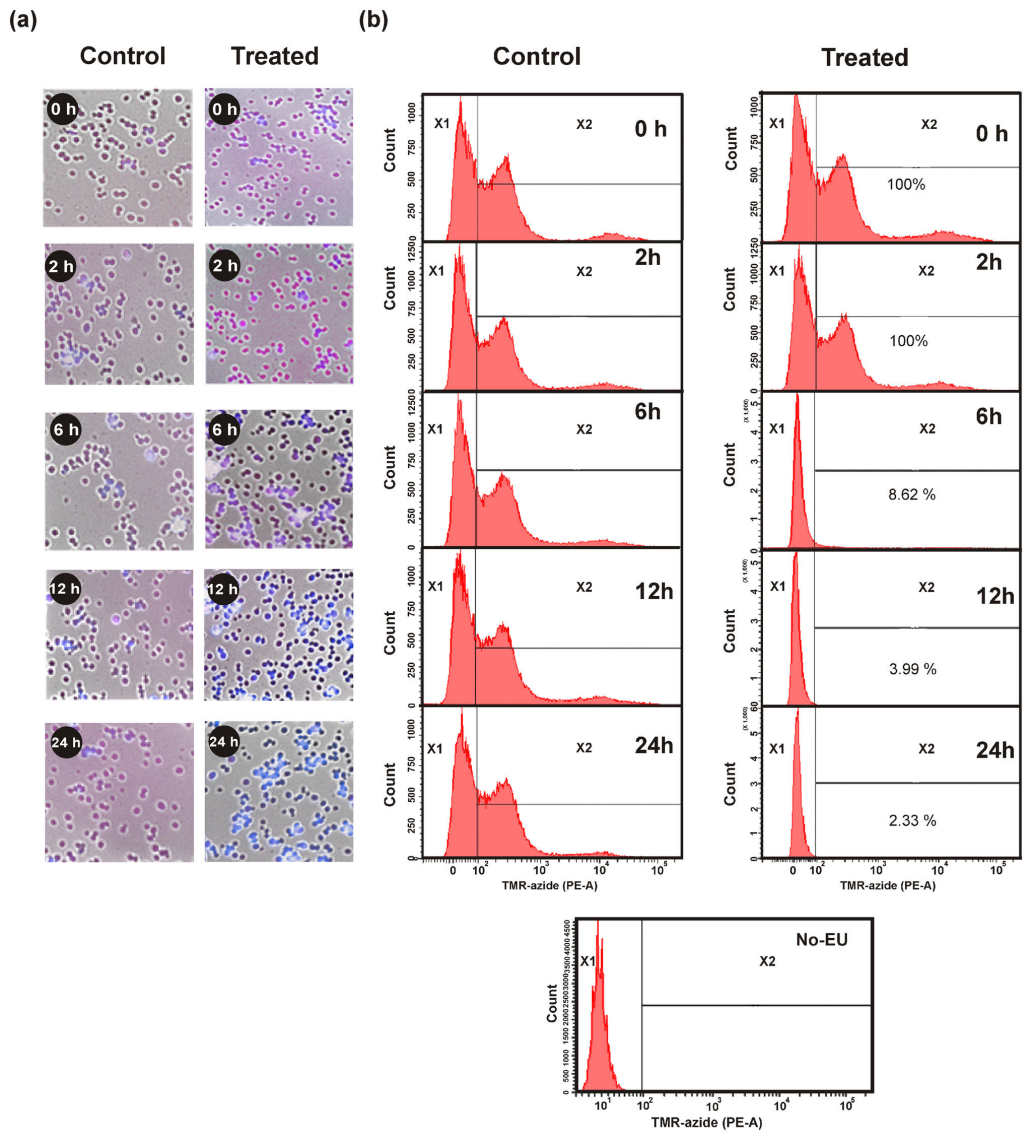
*In vivo* inhibition of transcription in *C. albicans* by MMGP1. (a) Confocal micrographs showing inhibition of transcription in *C. albicans* by MMGP1. The images are overlay of TMR-florescent azide (red), Hoechst 33342 (blue) and bright field micrographs of *C. albicans* cells. Intense EU staining (red fluorescence) was observed in nucleus after 2 h of treatment with MMGP1 and prolonged treatment of cells with peptide showed decrease in EU signal in the nucleus (b) Quantification of transcription inhibition in MMGP1-treated *C. albicans* by flow cytometry (X^2^-*C. albicans* cells showing TMR-A fluorescence i.e cells that are transcriptionally active).

The inhibition of transcription by MMGP1 was studied at varying peptide concentrations under *in vitro* conditions. Figure 4a & 4b shows the *in vitro* expression level of mouse β-actin gene in the presence of varying concentrations of MMGP1. No inhibition of transcription was observed at lesser peptide concentrations. The transcription reaction was found to be significantly inhibited (78%) at a higher peptide concentration (0.576 µM). The *in vivo* transcription inhibition by MMGP1 in *C. albicans* was studied based on the biosynthetic incorporation of EU into nascent RNA. At 2 h of incubation, intense EU staining (red fluorescence) was observed in the nucleus, which indicates the active incorporation of EU into the cells i.e., active transcription, whereas, EU signals in the nucleus dropped dramatically after 6 h of incubation (Figure 5a). Flow cytometry analysis also confirmed that transcription was not inhibited at 2 h of incubation, whereas only 8.62% and 3.99% of cells showed EU signals after 6 and 12 h of incubation, respectively (Figure 5b). Thus, the peptide inhibits the transcription *in vivo* in *C. albicans*.

### Endogenous ROS production

**Figure 6:**
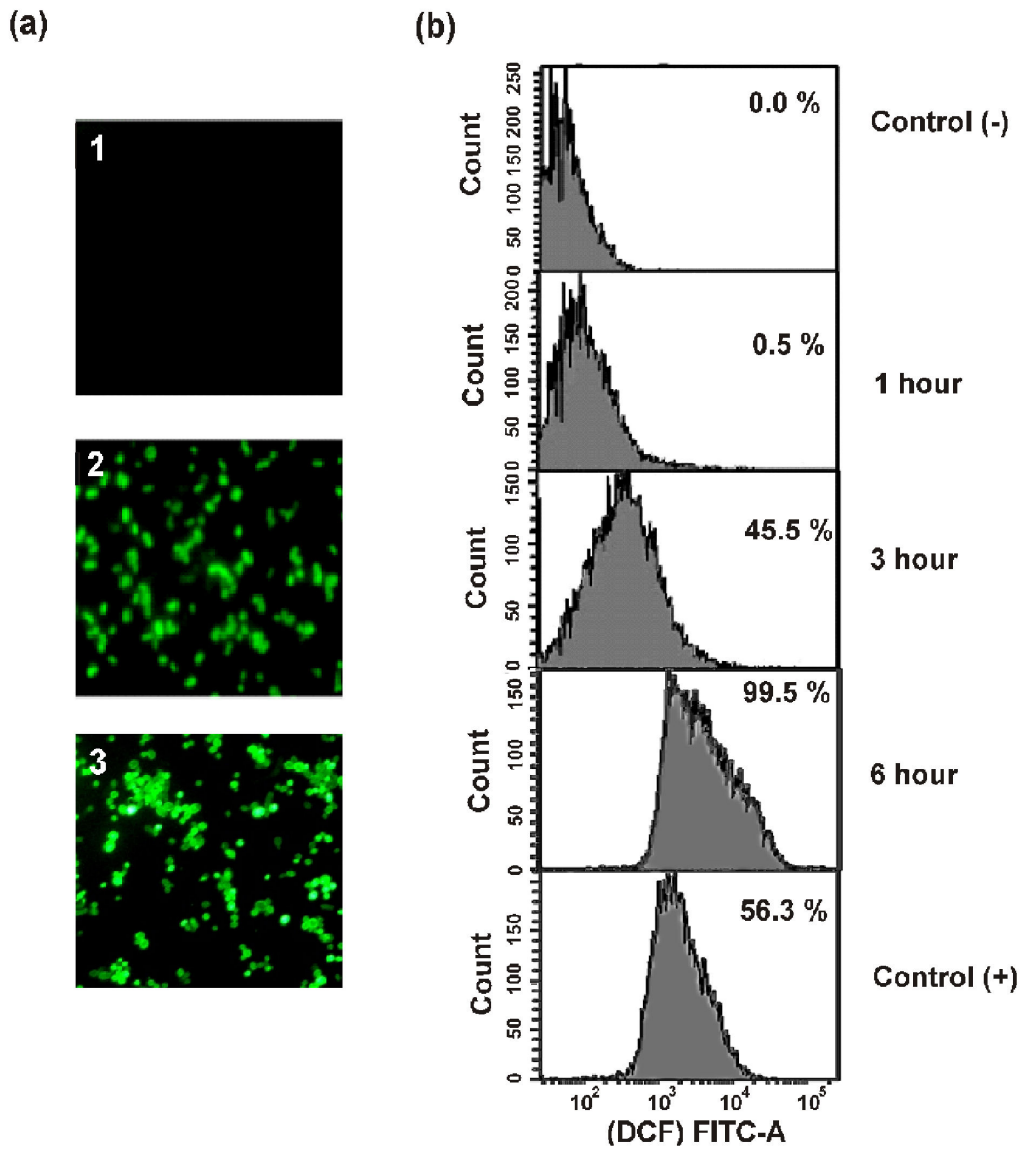
MMGP1 induced ROS production in *C. albicans*. (a) ROS induction in *C. albicans* cells treated with MMGP1. 1-C*. albicans* cells without MMGP1 (negative control panel); 2-C*. albicans* cells treated with MMGP1 for 6 h (Test panel); 3-*C. albicans* cells treated with H_2_O_2_ for 6 h (b) Time-scale measurement of intracellular ROS in MMGP1 treated *C. albicans* (0.57 µM) by flow cytometry. The fluorescence obtained with the cells treated with 1 mM of H_2_O_2_ serves as positive control and the cells without peptide serves as negative control.

MMGP1-induced endogenous production of ROS in *C. albicans* was analyzed by H_2_DCF-DA staining. H_2_DCF-DA is a cell-permeant and indicator of ROS, which is non-fluorescent until the acetate groups are removed by oxidation occurring within the cells. MMGP1-treated cells showed intracellular production of ROS, which was inferred by the DCF fluorescence of cells due to the oxidation of H_2_DCF-DA probe (Figure 6a). Quantification of endogenous ROS production in MMGP1-treated *C albicans* cells by flow cytometry revealed no significant increase in DCF fluorescence until 1 h of incubation with the peptide, whereas 45.5% of the cells showed DCF fluorescence after 3 h and more than 99% of the cells showed DCF fluorescence after 6 h of incubation with the peptide (Figure 6b).

### Viability changes on glutathione supplementation

**Figure 7:**
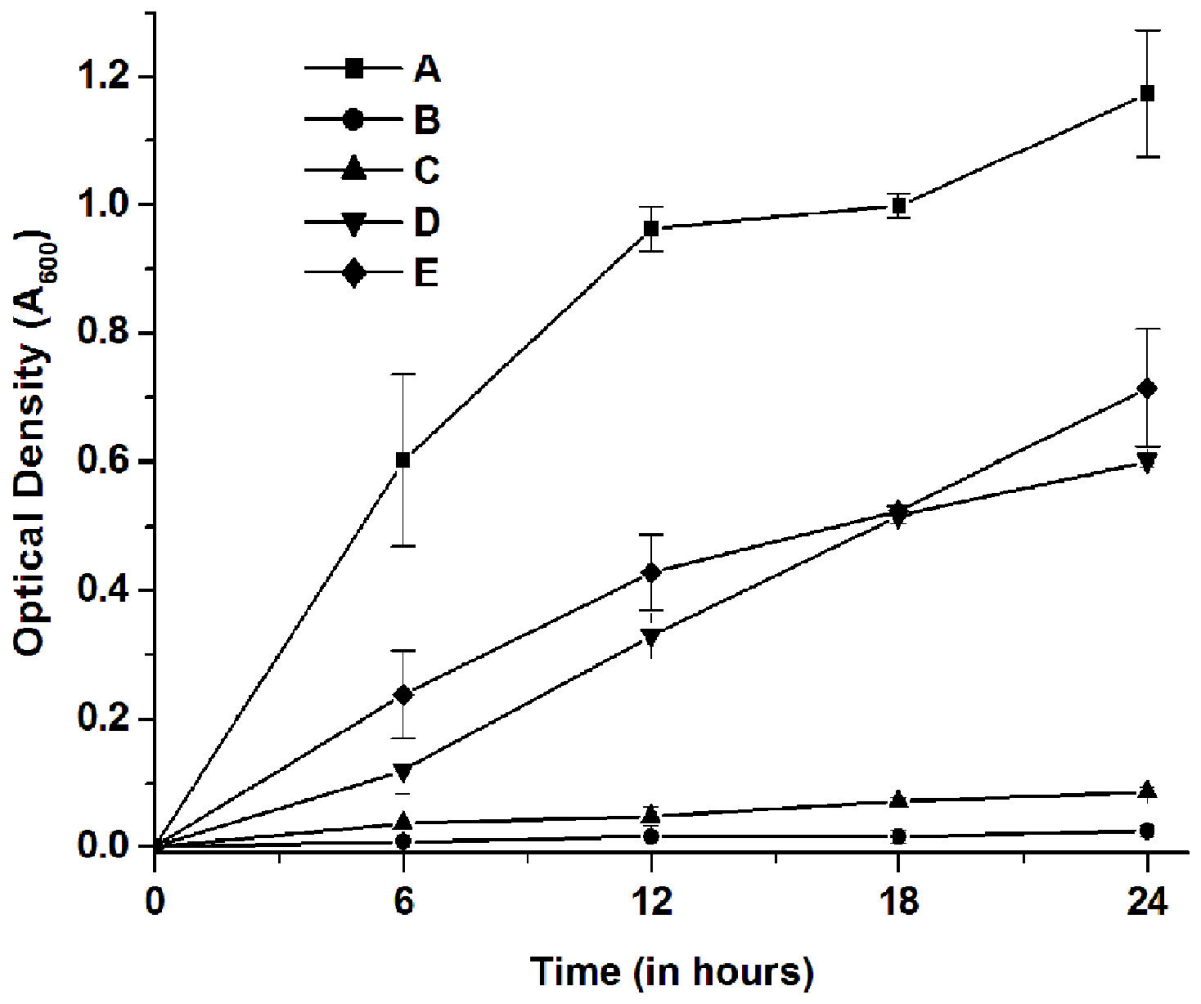
Effect of glutathione on viability of MMGP1-treated *C. albicans* cells. The cells were treated with peptide (0.57 µM) in the presence and absence of glutathione for 24 h. The cell density was measured at 600 nm for every 6 h interval. A-without peptide; B-with peptide; C, D, E-with peptide in the presence of 1, 10 and 50 mM glutathione, respectively.

The effect of MMGP1 on the viability of *C. albicans* cells after glutathione supplementation is shown in Figure 7. No significant increase in the growth of *C. albicans* cells treated with MMGP1 in the absence of glutathione was observed, whereas the cells treated with MMGP1 in the presence of glutathione showed an increase in cell viability as glutathione concentration increases.

### Oxidation of proteins and lipids

**Figure 8:**
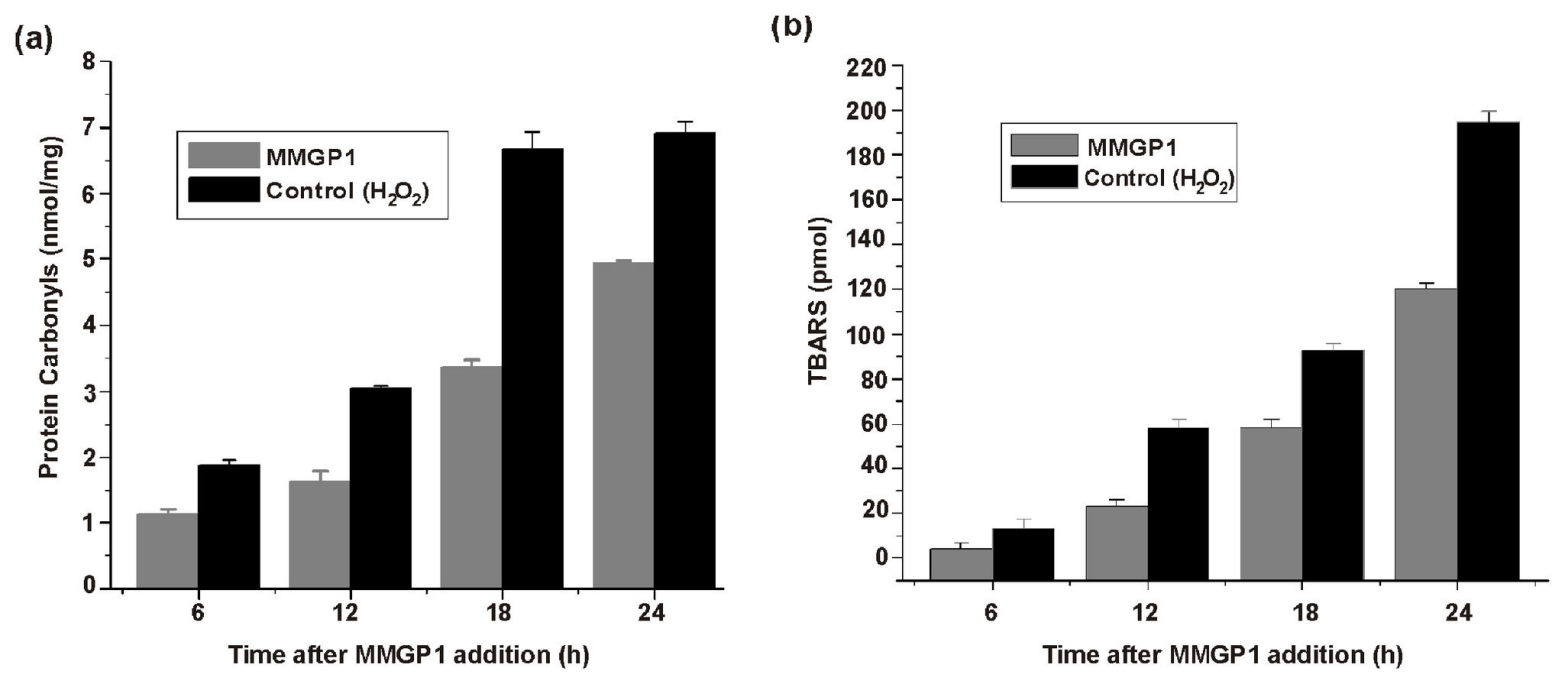
MMGP1-induced intracellular oxidation of proteins and lipids in *C. albicans*. (a) Time-dependent measurement of protein carbonyls in MMGP1 treated *C. albicans* cells by DNPH assay. (b) Time-dependent measurement of TBARS production in MMGP1 treated *C. albicans* cells by TBA assay.

An irreversible oxidation of proteins and lipids is the secondary effect of ROS produced within the cells. These deleterious modifications of proteins and lipids have lethal effects on target cells and lead to cell death. Therefore, the oxidation of intracellular proteins by ROS was studied by the examination of the cell lysates of *C. albicans* cells treated with MMGP1 at different time intervals. The results clearly indicated a time-dependent increase in the level of protein carbonyls in the treated cells (Figure 8a). In addition, the oxidation of lipids by ROS was investigated in MMGP1-treated *C. albicans* cells at different time intervals, and a time-dependent increase in production of TBARS was observed (Figure 8b). These consistent increases in the level of protein carbonyls and TBARS concentration at different time intervals indicate that the peptide induced oxidation of proteins and lipids.

### Disruption of mitochondrial membrane potential

**Figure 9:**
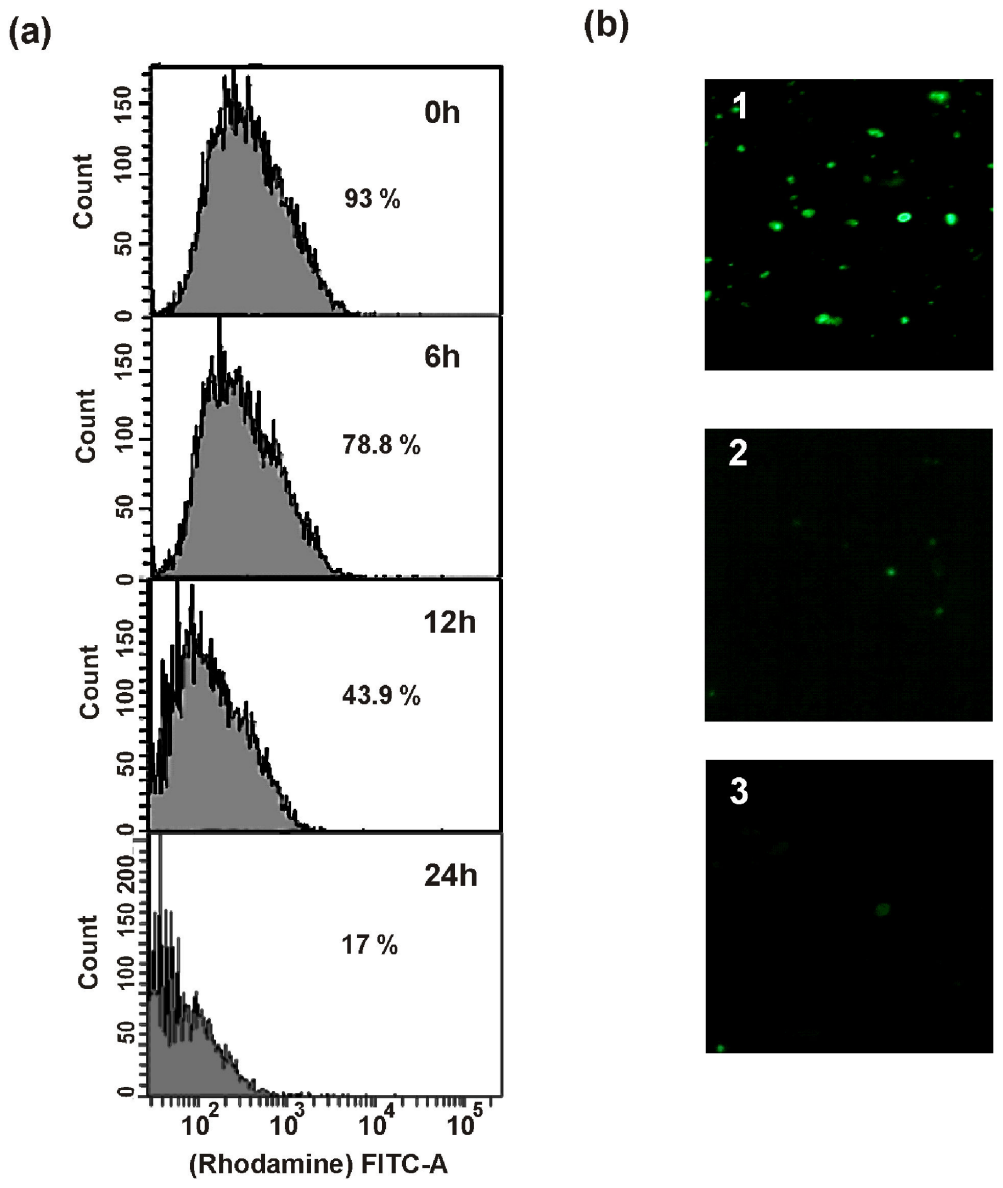
Mitochondrial membrane depolarization in MMGP1 treated *C. albicans* cells. (a) Measurement of mitochondrial membrane potential in MMGP1 treated *C. albicans* cells by flow cytometry (b) Measurement of inner mitochondrial membrane depolarization by MMGP1 in *C. albicans* cells. 1-mitochondria of *C. albicans* cells without treatment; 3-mitochondria of *C. albicans* cells treated with 1 mM H_2_O_2_; 2-mitochondria of *C. albicans* cells treated with MMGP1 for 24 h.

Dissipation of mitochondrial membrane potential in *C. albicans* cells by MMGP1 treatment was clearly evident from flow cytometry analyses. After 6 h of treatment with MMGP1, 73% of cells exhibited rhodamine fluorescence, whereas 43.9% of cells exhibited fluorescence after 12 h and only 17% of the cells showed rhodamine fluorescence after 24 h of treatment, which clearly indicate that the mitochondrial membrane potential is lost in 82% of the cells on treatment with MMGP1(Figure 9a). Figure 9b shows the NAO staining of mitochondria isolated from *C. albicans* treated with and without MMGP1. The intensity of NAO fluorescence diminished after 24 h of treatment with the peptide, suggesting the oxidation of inner mitochondrial protein cardiolipin, which could be attributed to the loss in mitochondrial respiratory potential.

### DNA damage

**Figure 10:**
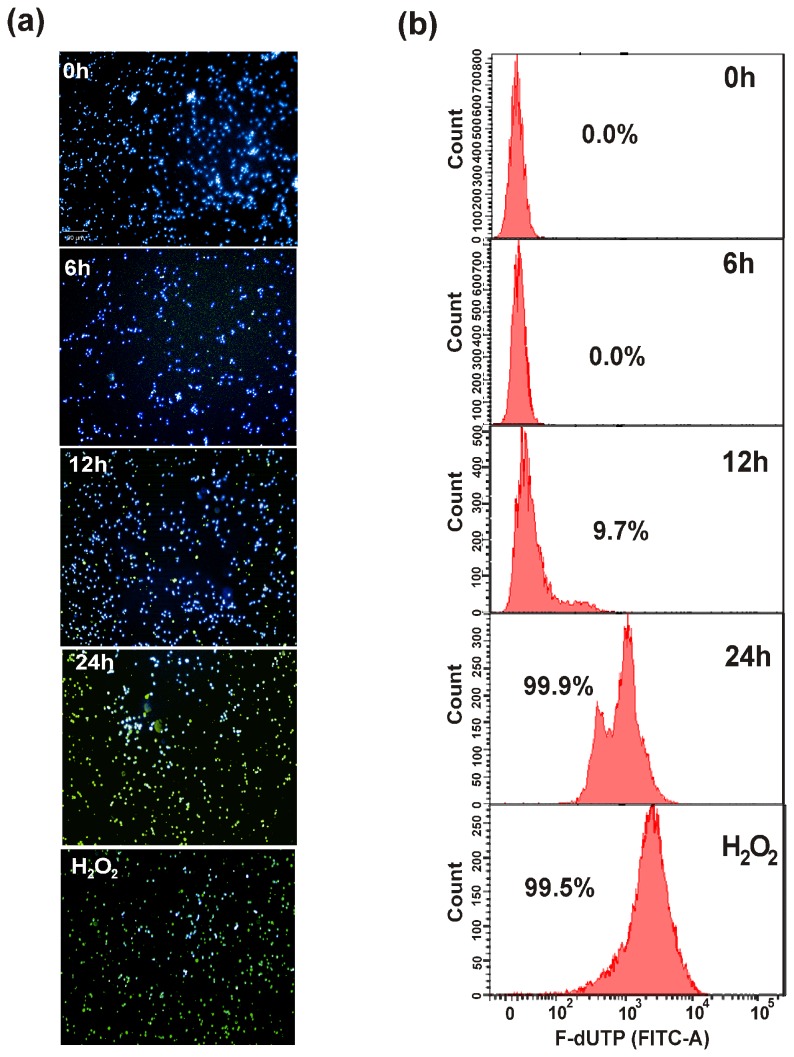
MMGP1 induced DNA damage in *C. albicans*. (a) Confocal micrographs of TUNEL stained *C. albicans* cells treated with MMGP1 for 0, 6, 12, and 24 h (20 x magnification). The images are overlay of TUNEL (green fluorescence), Hoechst 33342 (blue) and bright field micrographs of *C. albicans* cells (b) Flow cytometry analysis of TUNEL stained MMGP1-treated *C. albicans* cells at different time intervals (0, 6, 12, and 24 h). The cells treated with H_2_O_2_ was used a TUNEL positive cells.

The *in vivo* DNA damage in *C. albicans* was analysed by TUNEL staining. At 6 h of incubation with the peptide, no TUNEL positive nuclei (green fluorescence) were observed, whereas, the number of TUNEL positive nuclei increased after 12 and 24 h of treatment with the peptide (Figure 10a). FACS analysis of TUNEL-stained MMGP1 treated *C. albicans* cells are shown in Figure 10b. No TUNEL-positive cells were observed after 6 h of treatment, whereas, 9.7% and 99.9% of TUNEL-positive cells were observed after 12 and 24 h of treatment, respectively, which is an indicative of DNA damage induced by MMGP1 in *C. albicans*.

### Hemolysis

The hemolytic activity of the peptide against human erythrocytes is considered as the major index of toxicity toward human cells. Hemolysis was observed at a peptide concentration of 11.84 µM, which was relatively higher that the MIC of *C. albicans.*

## Discussion

Earlier, it was reported in our laboratory that the MMGP1 peptide induces cell death in *C. albicans* cells in a non-disruptive manner through energy-independent direct penetration mechanism [12]. Several antifungal peptides are translocated across cell membrane and are found inside the cell, wherein they can induce various inhibitory activities, disrupting normal cell functions primarily not linked with cell penetration [4]. In the present study, we investigated the mechanisms of antifungal action of MMGP1 in *C. albicans*. The MMGP1 showed a remarkable non-specific DNA-binding property *in vitro*. The use of SDS or trypsin to remove the peptide allows the direct analysis of the status of bound DNA in the peptide–DNA condensate [23]. Similarly, in this study, proteinase K was used to hydrolyse the peptide and the bound plasmid DNA was detected on the gel, which confirms the formation of peptide–DNA complexes. The peptide formed highly stable complexes with DNA, which is resistant to high ionic conditions (i.e., 0.3 M of NaCl) (data not shown) and nuclease activity. In addition, the peptide inhibited the transcription processes both under *in vitro* and in vivo conditions, and therefore was found to interfere with cellular transcription process once inside the fungal cell. This finding is consistent with the earlier report on the antimicrobial peptide, indolicidin, which induces cytotoxicity in the target cells by binding to DNA and inhibition of macromolecular synthesis [15].

The MMGP1 is also capable of inducing endogenous production of ROS in *C. albicans*, which could be attributed to its binding with DNA, thereby inhibiting the transcription process within the cells. The ROS production by MMGP1 triggers a cascade of events like protein carbonylation, lipid peroxidation, mitochondrial membrane depolarization and DNA fragmentation. An earlier report suggested that the production of reactive species at subtoxic concentrations regulate cell differentiation, proliferation, signal transduction and ion transport whereas excessive accumulation of ROS within the cells can cause damage to DNA, proteins and lipids, which lead to disorganization, dysfunction and damage of membranes and proteins. Specifically, the oxidation of lipids may cause impairment of membrane function, decreased fluidity, increased permeability to ions and potentially membrane rupture [24–26]. To examine the relationship between endogenous production of ROS and antifungal mechanism, we investigated the secondary effect of ROS production in *C. albicans* by MMGP1. The results suggested that the hyper-induction of ROS causes oxidation of proteins, lipids and induces DNA damage. Furthermore, the growth of the MMGP1-treated *C. albicans* cells recovered after supplementation with glutathione, suggesting that the endogenous production of ROS could be the major mechanism for antifungal action of MMGP1 in *C. albicans.*

Mitochondrial dysfunction due to depolarization of mitochondrial membrane is a marked characteristic of ROS-mediated cytotoxicity. Interestingly, the results of mitochondrial-mediated experiments suggested that the MMGP1 induced disruption of mitochondrial membrane potential in *C. albicans* by depolarization of inner mitochondrial membrane. This finding is consistent with the earlier report on the antimicrobial peptide, papiliocin, which induces cytotoxicity in *C. albicans* by disruption of mitochondrial integrity and consequent cell death [5]. Certain other antifungal peptides such as α- and θ-defensin, magainin, cathelicidin and histatin are known to disrupt the mitochondrial membrane potential and induce cytotoxicity in yeast cells [7,27].

To conclude, we have demonstrated the mechanism of the antifungal action of marine metagenome-derived cell-penetrating antifungal peptide, MMGP1, against *C. albicans*. The collective data presented in this study indicate that the peptide inside the target cell exerts antifungal action by binding with DNA and inhibiting transcription processes that trigger a series of events such as endogenous production of ROS; oxidation of proteins; lipid, DNA damage; and dissipation of mitochondrial membrane potential that eventually lead to cell death.
